# The relation between rhythm processing and cognitive abilities during child development: The role of prediction

**DOI:** 10.3389/fpsyg.2022.920513

**Published:** 2022-09-23

**Authors:** Ulrike Frischen, Franziska Degé, Gudrun Schwarzer

**Affiliations:** ^1^Department of Music, University of Oldenburg, Oldenburg, Germany; ^2^Music Department, Max Planck Institute for Empirical Aesthetics, Frankfurt, Germany; ^3^Department of Developmental Psychology, Faculty of Psychology and Sports Science, University of Giessen, Giessen, Germany

**Keywords:** rhythm processing, prediction, cognitive abilities, musical rhythm development, beat processing

## Abstract

Rhythm and meter are central elements of music. From the very beginning, children are responsive to rhythms and acquire increasingly complex rhythmic skills over the course of development. Previous research has shown that the processing of musical rhythm is not only related to children’s music-specific responses but also to their cognitive abilities outside the domain of music. However, despite a lot of research on that topic, the connections and underlying mechanisms involved in such relation are still unclear in some respects. In this article, we aim at analyzing the relation between rhythmic and cognitive-motor abilities during childhood and at providing a new hypothesis about this relation. We consider whether predictive processing may be involved in the relation between rhythmic and various cognitive abilities and hypothesize that prediction as a cross-domain process is a central mechanism building a bridge between rhythm processing and cognitive-motor abilities. Further empirical studies focusing on rhythm processing and cognitive-motor abilities are needed to precisely investigate the links between rhythmic, predictive, and cognitive processes.

## Introduction

Rhythm is a central component of music. The ability to perceive and produce rhythm enables people to make music. The first signs of these rhythmic abilities appear already in infancy (Winkler et al., 2009) and develop into adulthood (Thompson et al., 2015). In recent years, interest in research on rhythm processing, its development, and the connection to cognition has increased. Researchers found positive associations between rhythmic abilities and different cognitive abilities such as language, motor function, or executive functions (e.g., Anvari et al., 2002; Flaugnacco et al., 2014; Degé et al., 2015; Lesiuk, 2015; Slater et al., 2018; Trainor et al., 2018) with some even suggesting potential causal links (e.g., Moritz et al., 2013; Flaugnacco et al., 2015; Frischen et al., 2019; Lê et al., 2020). For example, it has been shown that music training that is highly based on rhythm processing cannot only improve rhythmic, but also benefit language abilities in typical developing children and children with developmental dyslexia (Moritz et al., 2013; Flaugnacco et al., 2015). Additionally, it has been revealed that rhythm-based music training can improve executive functions in preschoolers (Frischen et al., 2019; Williams and Berthelsen, 2019). However, although a lot of research has already been done on the association between rhythmic abilities and cognitive-motor abilities, the precise connections and underlying mechanisms are still unclear in some respects. To gain a better understanding of these relations, we will identify processes that are related to both rhythm processing and cognitive-motor processing. We propose that prediction could be such a process, building a bridge between rhythmic abilities and cognitive-motor abilities. Predictive processes are fundamental for human cognition and highly relevant for early cognitive development (Nagai, 2019). From the very beginning of life, children strive to identify regularities and contingencies in their physical and social environment based on which they make predictions (Köster et al., 2020). In the context of musical rhythm, predictive processes mean the anticipation of temporally ordered sound events which is usually formed with regard to the meter and the rhythm of a tone sequence. As predictive processes are not only of great importance in the context of rhythm processing, but rather play a significant role in a variety of cognitive processes, they can be considered as cross-domain processes that have the potential to connect rhythm and general cognitive-motor processing. Therefore, we hypothesize that prediction is particularly well suited to explain the link between rhythmic and cognitive abilities. In the following, we will focus on the development of rhythm processing, its link to cognitive and motor processing and we will describe the extent to which our hypothesis that predictive processing is partly involved in the relation between rhythmic and various cognitive abilities is in line with extant research findings.

## Rhythm processing and its development

Rhythm and meter are key components of music. According to Schulkind (1999) musical rhythm is defined as a serial pattern of variable tone durations in a melody that unfolds within a timing framework of a recurring pattern of beats, the meter. Meter organizes a series of beats into recurring patterns of stressed and unstressed beats. The beat (or tactus) is referred as one count of the meter and the most natural rate at which a listener might tap or clap to music. If you change the tempo of a rhythm (play a rhythm faster or slower), the relative proportions between the individual beats remain the same. Tempo is defined as the pace of music, or the rate at which beats unfold over time (McAuley, 2010).

In the literature we find several abilities that are described as rhythmic abilities, which are divided into rhythm perception and production abilities (Thackray, 1969; Bouwer et al., 2021). As perception abilities we count the ability to discriminate between rhythms and tempi, to memorize rhythms, or to detect the beat in a rhythmic sequence. Rhythm production abilities include tapping to a beat, reproduce rhythmic patterns, or movement to music (e.g., Thackray, 1969; Tierney and Kraus, 2015; Bouwer et al., 2021). Although studies found correlations between different rhythmic abilities, including correlations between rhythm perception and production abilities in adults (e.g., Keele et al., 1985; Fujii and Schlaug, 2013; Tierney and Kraus, 2015), no unitary rhythm ability could be identified. However, according to Tierney and Kraus (2015), rhythm memory and beat-based processing seem to be two central abilities that are dissociable from each other in adult samples. While rhythm memory is more dependent on auditory memory, beat-based processing requires the ability to detect regularities within a temporal sequence, which can be related to an underlying meter [Ozernov-Palchik and Patel, 2018; Note: Beat-based processing is not a uniform term. For the term “beat-based” we also find terms like “metrical” (Essens and Povel, 1985) or “metric simple” (e.g., Grahn and Brett, 2009) in the literature; in this article we stick to the term “beat-based processing”]. Here, we focus on rhythm processing in terms of beat-based processing as predictive processes can be assumed to be a central component of this ability, what we will illustrate in the following sections. Moreover, previous studies already indicated that this could be a bridging principle in the relation to cognitive-motor-abilities (Ozernov-Palchik and Patel, 2018).

Similar to the question of how many and what rhythmic abilities exactly exist, the development of rhythmic abilities in humans is not yet fully explored so that a detailed consideration of the development of rhythmic skills has not yet been done. However, there is a lot of literature on certain time periods of rhythmic development with a focus on infancy and childhood up to about 7 years of age. In infancy, motor control is still under development. Therefore, rhythm perception abilities are ahead of rhythm production abilities. At the perceptual level, first signs of rhythmic ability occur very early. Evidence has been found indicating that few days old newborns already show specific responses in the electroencephalogram (EEG) to changes in sound-durations, which are similar to those EEG-responses in adults suggesting that infants are sensitive to changes in sound durations (Kushnerenko et al., 2001). Moreover, it has been revealed that newborns can detect repetitive sound patterns (Stefanics et al., 2007) and that they are sensitive to omissions of the downbeat within presented sound sequences (Winkler et al., 2009). These findings indicate that the predispositions for more complex rhythmic abilities are already present in newborns. Further studies have shown that those precursor rhythmic abilities continue to develop within infancy. In a study by Phillips-Silver and Trainor (2005) it was demonstrated that infants’ encoding of a meter could be influenced by moving them, indicating that they can already distinguish between a double and a triple meter. Moreover, a recent EEG-study by Flaten et al. (2022) showed that 6-month-old infants can extract information about meter from auditorily presented stimuli and transfer it to an auditorily presented ambiguous rhythm. Additionally, results revealed that infants with musically experienced parents showed larger EEG amplitudes indicating that parental musical background influences music perception in infants. Similar results have been reported in a study by Cirelli et al. (2016) with 7- and 15-month-old infants. Their results revealed that infants’ music experience and parents’ musical background influenced EEG amplitudes corresponding to beat and meter. In sum, results of both studies suggest that already in early development individual differences in music experience and parental musical background affect rhythm processing and music processing in general.

As already mentioned, rhythm production abilities develop later from early childhood on. A study with 5- to 37-month-old children indicated that children’s spontaneous motor tempo (SMT) becomes faster with age, because they get better in the ability to make repeated, targeted movements. Additionally, it seems that the SMT is related to the step rate of their parents, suggesting that early rhythm is set by the vestibular stimulation from parental carrying (Rocha et al., 2021). In contrast to freely producing rhythms such as measured by the SMT, synchronizing movements to a rhythm is a more complex skill: During infancy, children are not able to fully synchronize their movements to a musical beat, although they move their arms and legs in response to music (Fujii et al., 2014) and can adapt their movements to tempo changes to some extent (Zentner and Eerola, 2010). A more recent study on children’s drumming revealed that synchronization abilities start to develop around the age of 2 years, but only in a tempo which is close to the children’s own spontaneous drumming tempo (Yu and Myowa, 2021). The ability to adapt to a slower tempo was only shown in older children from 30 months on. Overall, the study showed that synchronization abilities improved from 18 over 30 to 42 months of age. These results fit well to the results of previous studies, showing that synchronization abilities are still under development in early childhood (e.g., Drake et al., 2000; Provasi and Bobin-Bègue, 2003; McAuley et al., 2006; Kirschner and Tomasello, 2009). For example, the study of McAuley et al. (2006) revealed that children around the age of 2.5 years can manage to tap in synchrony with an isochronous (temporally equidistant) beat, which is close to their own spontaneous drumming tempo, but fail when they are asked to adapt their tapping to different tempi. In contrast, 4-year-old children performed significantly better in adjusting their tapping to different tempi. Further results of this study found in the appendix and reported by Repp and Su (2013) show that 4- and 5-year-olds are still not good at synchronization while 7-year-olds perform almost on an adult level. Similarly, Drake et al. (2000) reported that 4-year-olds already show the ability to synchronize to different tempi and stimuli but that this ability improves further with age.

Taken together, findings from the literature indicate that rhythmic abilities occur already early in life and develop during childhood. While newborn infants are already sensitive to sound durations and rhythmic variations, rhythm production abilities, and especially the ability to synchronize to a beat, which highly relies on beat-processing occurs earliest in young childhood at around 2 years and improves with age.

## Rhythmic abilities and cognitive-motor abilities

Previous research showed that rhythmic abilities do not only develop rapidly during early development, but that they are also connected to the development of non-musical abilities such as in the cognitive and motor domain. In this section, we will address this research and focus on the relation between rhythmic abilities and three specific areas within cognitive-motor abilities: language ability, motor skills and executive functions. These relations serve as examples based on which we will develop our hypothesis on predictive processes as a bridge between rhythmic abilities and those three cognitive-motor areas. In the literature we find mainly correlative studies suggesting positive associations between measures of rhythmic abilities and cognitive-motor abilities: It has been shown that rhythmic abilities are associated with language related abilities such as reading, or precursors of reading ability (Anvari et al., 2002; Thomson et al., 2006; Thomson and Goswami, 2008; Huss et al., 2011; Moritz et al., 2013; Tierney and Kraus, 2013; Flaugnacco et al., 2014; Degé et al., 2015; Tierney et al., 2021; Bégel et al., 2022; for a review see, e.g., Ladányi et al., 2020), executive functions (Tierney and Kraus, 2013; Lesiuk, 2015; Slater et al., 2018), and motor abilities. The latter indicated by the finding that populations with motor disabilities show poorer rhythmic abilities than typical participants (Whitall et al., 2008; Grahn and Brett, 2009; Roche et al., 2016; Trainor et al., 2018). Moreover, there are also training studies suggesting causal relationships between rhythmic activities and language (Flaugnacco et al., 2015; Lê et al., 2020), rhythmic activities and executive functions (e.g., Frischen et al., 2019; Williams and Berthelsen, 2019), and rhythmic activities and gait in Parkinson’s disease (e.g., Thaut et al., 1996; for a review see Nombela et al., 2013). Theoretically, it would be plausible that such effects could also occur in the other direction, from the training of cognitive-motor skills to rhythmic skills. However, we do not know of any studies on this topic so far.

In the following, we will describe some of these studies on the relation between rhythmic abilities and the three non-musical areas of language ability, motor skills and executive functions in detail to analyse and show that one particular aspect of rhythmical experience, beat-based processing, is particularly related to these non-musical abilities.

A study conducted with infants underlined the strong link between musical rhythm and language processing. In a training study, Zhao and Kuhl (2016) presented short waltz-like musical pieces to 9-month-old infants in 12 training sessions to familiarize them to the temporal structure of a triple meter. The infants were tested before and after the training sessions with respect to their neural responses to violations of this temporal structure using musical and non-native syllable-like sequences. The authors found that infants in the music-intervention group showed a larger neural response to a violation of the triple meter in musical sequences and, crucially, also in the speech-like stimuli compared to infants in a control group who did not participate in the music intervention. The detection of the temporal structure of the musical pieces has thus generalized to the detection of a comparable sequence of speech-like stimuli.

Regarding older children, the study of Degé et al. (2015) found out that rhythm perception, and rhythm production tasks are related to measures of phonological awareness in pre-schoolers. Phonological awareness refers to the ability to recognise, analyse and manipulate sounds in oral language (Stahl and Murray, 1994; Lonigan, 2006) and is an important predictor of reading and writing abilities (e.g., Marx, 2007). In a correlational approach, they tested different measures of musical ability and phonological awareness while controlling for IQ and socioeconomic status in pre-schoolers. First, results showed that several musical abilities were related to measures of phonological awareness. However, after controlling for the first type of error, only the music production tasks related to rhythmic skills still showed a significant association with measures of phonological awareness. Flaugnacco et al. (2014) reported similar results in a sample of children diagnosed with dyslexia. Their findings showed that measures of rhythmic abilities (tapping to a metronome, rhythm reproduction, and meter perception) are related to reading skills. Additionally, in a following training study it has been shown that a rhythm intervention can improve reading abilities in children with dyslexia (Flaugnacco et al., 2015). Ozernov-Palchik et al. (2018) investigated the previously reported relation between rhythmic abilities and literacy skills in more detail and pursued the question whether there is a specific feature in rhythm that is especially linked to literacy skills. A specific characteristic of most rhythms is that they have a regular underlying structure of recurring beats, what they term as “beat-based.” Beat-based rhythms can be grouped into equal time units whereas in non-beat-based rhythms the beats cannot be grouped in equal time units. Since there is a regular underlying structure in beat-based rhythms, the occurring beats can be anticipated. In a correlational approach, the authors tested beat-based as well as non-beat-based rhythm perception in a sample of 5- to 6-year-old children and found relations between both the beat-based and the non-beat-based rhythm task and different early literacy skills. However, they found especially the beat-based task being a unique predictor of one measure of early literacy skills (letter-sound knowledge) above general cognitive abilities, phonological awareness, and non-beat-based processing.

Taken together, research on rhythmic abilities and language abilities shows significant associations that occur already in infancy and could be demonstrated in typically developing children and children with dyslexia even with a first indication of a causal link. Additionally, first evidence emerges that especially beat-based processing plays a significant role in the relation between rhythm abilities and language abilities.

Regarding the association between rhythm abilities and motor function, studies revealed that children and adults with motoric disorders show difficulties in rhythm processing as indicated through studies with children with Developmental Coordination Disorder (DCD; e.g., Whitall et al., 2008; Roche et al., 2016) as well as adults affected by Parkinson’s disease (PD, a neurodegenerative condition including symptoms of problems in walking and gait; Knutsson, 1972; Grahn and Brett, 2009; Nombela et al., 2013). Whitall et al. (2008) showed that children with a diagnosis of DCD have poor rhythmic skills. In their study they compared children diagnosed with DCD with gender and age-matched typical controls as well as with typical adults in a finger-tapping paradigm. The results demonstrated that children with DCD are broadly able to match their tapping to the different metronome tempi. However, children with DCD have particular problems to match the beat in slow tempi. Moreover, the tapping of the children with DCD was more variable compared to age-matched controls and adults. While adults mostly show to be a bit before the beat, children tend to tap behind the beat. The children with DCD, however, did not show any consistent relation with the beat. These results indicate that children with DCD have problems in identifying and anticipating the single beats within a rhythm sequence, so that they seem to have difficulties in beat-based processing. Similar results have been revealed from studies with older people diagnosed with PD indicating that people with PD have poorer rhythmic abilities compared to typical controls (e.g., Grahn and Brett, 2009; Hsu et al., 2022). For example, Grahn and Brett (2009) tested older people affected by PD and typical controls in a rhythm discrimination task similar to the task described in Ozernov-Palchik et al. (2018) consisting of two conditions: a beat-based and a non-beat-based condition. The results revealed that people diagnosed with PD do not show differences in both conditions of the task, while typical controls showed a better performance in the beat-based task compared to the non- beat-based task. Moreover, older adults affected by PD performed worse in the beat-based task compared to typical controls. The results indicate that people affected by PD also have problems in the detection and anticipation of the beat structure indicating problems in beat-based processing. In sum, the reported studies on the relation between rhythmic abilities and motor function also indicate that beat-based processing seems to be a fundamental ability which is linked to motor skills.

In addition to associations between rhythmic abilities and more specific non-musical abilities such as language and motor function, associations between rhythmic abilities and general cognitive abilities, such as executive functions, have also been revealed: Frischen et al. (2019) found out that a rhythm-based music intervention can enhance inhibition (a measure of executive functions) in pre-schoolers. In a randomized controlled training study, children from different kindergartens received a 6-months rhythm-based music intervention, a pitch-based music intervention, or a sports intervention three times a week. Before and after the intervention children’s executive functions were assessed. The results showed that only the rhythm group improved significantly from pre- to post-test in inhibition, suggesting that rhythm training can improve inhibition skills in young childhood. Further measures of executive functions (working memory, flexibility) were not significantly affected by any training. While this study suggests that rhythmic practice promotes inhibition, it leaves open which rhythmic abilities exactly were trained and how they are linked to inhibition. Related to this issue the study of Tierney and Kraus (2013) indicated that rhythm production ability, measured by tapping to a beat, was positively correlated with inhibition in adolescents. In this study, tapping performance was assessed in two conditions: tapping to the beat (paced condition) and tapping in silence (unpaced condition). Inhibition was measured with a test including an auditory and a visual condition. The results showed that tapping to the beat (paced condition) was positively correlated with both auditory as well as visual inhibition. However, the unpaced tapping condition was not correlated to inhibition in any way. Moreover, it has been found that less tapping variability was associated with better performance in the auditory and the visual inhibition task. These results have been confirmed in a following study with a similar design revealing that less variable drumming was positively associated with inhibition in young adults (Slater et al., 2018). Since tapping or drumming to a beat is highly dependent on beat-based processing, it seems that especially this rhythmic ability is of great importance in the relation to inhibition as one measure of executive functions, which is in line with the previously reported findings on language abilities and motor function. However, since the reported studies did not report on relations between beat-based processing and further executive functions apart from inhibition, it remains unclear whether there is a specific relation solely to inhibition or whether we can assume a relation between beat-based processing and executive functions in general.

Taken together, there is a substantial amount of literature suggesting correlations between rhythmic and cognitive-motor skills. In addition, there are individual studies that suggest causal relationships. Interestingly, studies on the relation between rhythmic abilities and non-musical abilities from all three reported areas suggest that the ability to extract regularities from rhythms, such as in beat-based processing (e.g., Ozernov-Palchik et al., 2018) is a highly relevant aspect of rhythmic experience that is connected to cognitive-motor abilities.

## Processes involved in the association between rhythmic abilities and cognitive-motor abilities

Despite the reported findings indicating a close association between beat-based rhythm processing and cognitive-motor abilities, it is still unanswered how these rhythm processes possibly exert an effect on the above-mentioned non-musical abilities. We propose that one possible process involved in this relation could be the process of making predictions, which is a key process in beat-based rhythm processing and cognitive-motor processing as well.

The ability to recognize a beat-based rhythm requires that an internal representation or model of the rhythm with the corresponding beats has been generated. Such an internal representation is necessary as it provides the basis for anticipating the upcoming beats. Thus, the formation of expectations and the reduction of expectation errors represent fundamental processes in the perception and recognition of a rhythm. The rhythm in a piece of music “plays” with predictions to generate and convey tension and tension release through the violation and fulfilment of expectations in musical sequences. The predictive character of a rhythm also becomes particularly obvious when a rhythm is to be played or clapped synchronously. Imagine this process would be a reactive instead of anticipatory process, then one would always be a little behind the beat and a precise timing would not be possible. Thus, synchronous production of a rhythm is only possible because we are anticipating the beat we are about to produce. Therefore, prediction can be understood as an inherent component of perceiving and producing a rhythm.

However, prediction is not only a key process of beat-based rhythm processing but also a key process of human perception and cognition in general. Continuously making predictions is indispensable and vital for survival. Making predictions can be considered as a working principle that aims to constantly adapt mental representations of the environment to its requirements by making predictions and learning from prediction errors. This in turn leads to a minimizing of prediction errors and to an increase of successful interactions with an ever changing environment (see Köster et al., 2020). The origin of research on predictive processes can be traced back to basic motor learning principles as already described by Helmholtz (1867) or see (e.g., Schubotz, 2015) which have recently been transferred as a basic learning principle of the human brain (e.g., Friston, 2005, 2010). Viewed from this theoretical framework, predictive processes occur on various, hierarchically organized levels: from basic, often automatic motor responses to controlled, higher reasoning. Feedback about prediction errors is sent back to the levels in the hierarchy that are involved in the prediction process to adjust existing predictions and internal models. This can then lead to changes on the motor up to the cognitive level (see Köster et al., 2020). Thus, predictive processing is not a single cognitive ability, but a cross-domain working principle that encompasses perception, motor skills (action), and cognition.

Regarding predictive processes during development, previous research has mainly focused on such processes outside the domain of music or rhythm processing. Here, it has been demonstrated that from early infancy on, children have the strong motivation to detect regularities in their environment using statistical information in stimulus sequences from which they form predictions (Bulf et al., 2011). Such statistical information, e.g., specific frequencies, redundancies, or transitional probabilities of stimuli in a sequence can be found in almost all natural events in our auditory and visual environment and can be perceived without any instructions or feedback. Perceiving statistical information is considered a mandatory process of human information processing (e.g., Gómez, 2017) which allows the detection of patterns in structured inputs that can also serve as a basis for predicting subsequent events. Evidence of this mechanism was provided in the seminal work of Saffran et al. (1996) on the statistical learning of continuous speech patterns in 8-month-old infants, which led to an explosion of research on this topic. This body of research showed that infants and children recognize statistical information in a variety of sensory domains, not only in language and other auditory stimuli, but also in sequences of visual and haptic stimuli (e.g., Kirkham et al., 2007; Fassbender et al., 2014; Aslin, 2017).

Previous research on non-musical predictive processes during development is also based on the idea that children’s predictions are more and more controlled by their sensorimotor experiences. This basic idea originates in Piaget’s work on children’s sensorimotor development (Piaget, 1952), and has been convincingly confirmed by Nagai (2019) work on predictive learning. Nagai proposes two modules which represent the architecture of infant predictive learning. The first module comprises the sensorimotor system which has the role of executing actions, interacting with the environment, and recording the resulting sensory feedback from the environment. The second module represents the so-called predictor, which comprises the internal model of the sensorimotor system. The aim of the predictor is to accurately simulate the sensorimotor system by learning to minimize the so-called predictive error. Nagai assumes that an infant’s predictor needs to develop and constantly improves with increasing sensorimotor experience which is in agreement with the conception of so-called forward models of motor control in children and adults. These models also emphasize the extremely close connection between motor performance and prediction. Forward models are used by the Central Nervous System (CNS) to internally simulate the behavior of the motor system in planning, control and learning (Wolpert and Miall, 1996). When a motor signal from the CNS is sent to the periphery, a copy of this motor outflow (i.e., reference copy) is generated. This reference copy inputs to the internal model which can estimate the sensory consequences of the motor command, thus generating the predicted sensory feedback. This forward mechanism is used to anticipate the sensory effects of movement. Thus, the sensory consequences of self-generated movements can be accurately predicted. This mechanism clearly shows the high extent to which motor behavior and prediction are interwoven with each other. In our own research we could demonstrate how increasingly correct predictions in infancy correlate with the increase in infants’ motor experience (e.g., Schwarzer, 2014). The prediction of visual–spatial object relations was improved in infants with advanced crawling and manual object exploration skills, compared to infants with low motor skills. In particular, our results suggest that infants with different types of locomotion and manual object exploration experiences differ in their visual processes based on which they generate their visual–spatial predictions (Gerhard-Samunda et al., 2021; Kelch et al., 2021). It can therefore be stated that the developing motor system acts as a control mechanism which promotes correct predictions or reduces prediction errors. Motor experiences allow children to identify regularities in their environment based on which they generate internal models of their environment from which they form predictions leading to increasingly lower prediction errors.

Another line of research on the development of general predictive processes focuses on the idea that children base their predictions on their increasing prior knowledge. Stahl and Feigenson (2015), for example, demonstrated that 11-month-old infants responded to a violation of their expectations regarding their physical core knowledge, and thus showed that the infants had made a particular prediction based on that knowledge. A study by Senju et al. (2011) provided evidence that children’s predictions based on their own theory of mind enable them to attribute mental states to others. Thus, it is obvious that all the acquired knowledge of children forms an essential basis for their predictions. In addition, especially in older children, metacognitive knowledge, knowledge about one’s own cognitive processes, can also serve as a basis for improving predictions. It can be assumed that applying predictions to one’ s own thinking in the sense of comparing learning goals to what has been achieved can improve higher-level cognitive outcomes. It is thought that predictive internal representations of the future are constantly compared with the actual perceived outcome of internal mental and external events. In this respect, making predictions allows to learn from previous experiences, a process that can be applied to various domains.

Overall, it can be summarized that children’s general predictive processes continuously improve with age and are mainly based on the detection of regularities in terms of statistical information, sensorimotor experiences, and acquired knowledge from which they generate their predictions. This is in line with Köster et al. (2020) who argued that a predictive-processing framework may provide a unifying umbrella of these at first sight unrelated cognitive processes. They considered prediction of future events as a general, early learning goal which is coupled with the ongoing motivation to reduce experienced uncertainty and to extract predictive structure from physical and social events. So far, only little research exists on the development of predictive processes in rhythm processing. However, we assume that the regularities from which predictions are formed in the course of general cognitive development can also be used for the formation of predictions in the processing of rhythms. For example, Trainor et al. (2003) and Trainor (2012) could show that even infants detect deviations in regular sequences of tone lengths and thereby showed their recognition of the temporal statistic within a rhythmical stimulus sequence. Interestingly, Markova et al. (2019) provided evidence that infants can detect rhythmical information in social interactions such as affective touch or singing with adults. They assume that entrainment (the process when neural oscillations couple to an external rhythm) to these social rhythms underlies the formation of interpersonal synchrony and thus stimulates reciprocal interactions between infants and their caregivers (see also Pereira et al., 2019; Nguyen et al., 2021). With respect to the role of sensorimotor experiences in prediction processing, Phillips-Silver and Trainor (2005) impressively demonstrated that bouncing movements influenced the encoding of meters in infants indicating a close connection between sensorimotor stimulation and temporal processing. Regarding the impact of prior knowledge on rhythm-based predictions, Vuust and Witek (2014) showed that adults’ rhythmic predictions are inferred from their previous musical experience and described that the processing system is always in a relation between bottom-up and top-down processes. They provided evidence that, for example, during syncopation – a rhythmic structure that violates metric expectations – the listener’s previous musical training determined the accuracy of the participant’s predictions.

Thus, we assume that children apply their general drive to make predictions about rhythmic events as well, using similar cognitive processes as they do for events outside the music domain and hypothesize that predictive processing in the general cognitive domain and in the domain of processing rhythms develops along similar types of regularities. We also presume that predictive processes from the rhythmic and cognitive-motor domains can influence each other, as the basic striving to make predictions can be similarly manifested in both domains. Nevertheless, it could be speculated that at least for children relatively simple rhythms or meters are one of the best examples by which predictions and their confirmation can be experienced, which is why they could have a unique, prediction-stimulating effect.

## The role of predictive processes in the relation between rhythm and cognitive-motor processing

As predictive processes are crucially involved in the domain of beat-based rhythm processing and cognitive and motor processing, we suppose that predictive processes could serve as mechanisms which could partly explain the association between rhythmic processing and non-musical cognitive-motor abilities. We believe that predictive processes in rhythm processing can stimulate similar processes in the cognitive-motor domain and thereby have the potential to build a bridge between the domains. In the following, we will describe such a potential bridge in more detail using findings from some of the previously mentioned studies. For example, the study by Zhao and Kuhl (2016) demonstrated that infants detected the metrical statistic in a musical piece and transferred it to the same metrical statistics, now however presented in speech-like stimuli and were able to make predictions on such a statistical regularity. These results demonstrate that already in infancy predictive processes based on the recognition of statistical regularities can be used from one into another domain. Moreover, we assume that prediction might be the process that builds the bridge between rhythm processing and linguistic abilities. In a study with pre-schoolers, Ozernov-Palchik et al. (2018) found out that especially the processing of beat-based rhythms is linked to precursors of reading abilities. Interestingly, Ozernov-Palchik and Patel (2018) describe that prediction is involved in beat-based processing and how this is linked to literacy abilities. They point out that beat-based rhythms consist of recurring temporal statistics that can be predicted and that also language, or literacy skills in specific, are based on predictive processes. While in beat-based rhythms, the process of prediction lies specifically in being able to predict future musical rhythmic events based on the rhythmic statistics that are emitted, in the case of literacy skills, the prediction process lies more in being able to make predictions about future linguistic material on the basis of the linguistic structure (e.g., phonological structure, syntactic structure). For example, through statistical learning, children gain knowledge about frequently occurring phonetic combinations and can make predictions about how words are put together from phonemes. The same applies to the translation of phonemes into graphemes: Through statistical learning, children gain knowledge about how phonemes are often transferred into graphemes. Based on this knowledge, they can make predictions about how words are going to be written down. Studies found that in children with developmental dyslexia predictive rhythm processing is disturbed, which is not only reflected in poor rhythm perception and production ability (e.g., Flaugnacco et al., 2014; Bégel et al., 2022) but also on a neural level: Children with developmental dyslexia show atypical neural rhythmic entrainment during beat perception and production (Colling et al., 2017). Thus, the results of the studies with typical developing children and with children with developmental dyslexia are in line with our hypothesis that prediction may be involved in the rhythmic-language relation and may build a bridge between the two abilities.

Also, studies that focused on the relation between rhythmic processing and motor abilities are in line with the idea that prediction could be a bridging process: In the previous section, we already explained the strong link between the development of the motor system and predictive processing. This strong link becomes also evident through the above-mentioned example of adults and children living with motor disabilities such as in DCD or PD. As reported above children with DCD and older adults suffering from PD both have particular problems in processing beat-based rhythms (Grahn and Brett, 2009; Roche et al., 2016), which – as already explained – heavily rely on predictive processes and especially when a rhythmic motor skill is required on generating effective forward models. For example, the study of Whitall et al. (2008) revealed that children with DCD have poorer tapping performance compared to typical children and adults. The finding that the tapping of children with DCD is more variable without a consistent relationship to the beat indicate that impaired forward models with correspondingly impaired prediction processes account for the more variable tapping behavior. Predictive processes are especially essential for motor control. Without an internal model of a planned motor action and the prediction of the outcome of a movement, it is not possible to adapt, or correct rapidly this movement sequence, if required (e.g., to correct tapping behavior when it is not perfectly matched to the beat, or when the beat is changing). In fact, it has already been proposed that children with DCD have problems in motor control due to impaired forward models and therefore impaired prediction of motor sequences. Interestingly, it has been found, that these impaired predictive processes in DCD are not only found in relation to motor function, but also in relation to other cognitive domains (Opitz et al., 2020). This finding supports the idea that predictive processing can function as a cross-domain mechanism that is relevant for several cognitive-motor tasks and can potentially provide a link between rhythm processing and cognitive-motor processing. Regarding children with DCD, it can be assumed that the weak tapping performance can be explained through a general impairment of predictive processes that influence both, the exact anticipation of the beats and the execution and adaptation of movements.

Lastly, predictive processes could serve as a linking process in the relation between rhythmic processing and inhibition (as one measure for executive functions). As reported above, the results by Frischen et al. (2019) demonstrated an enhancement in children’s inhibition skills after a rhythm training intervention and the results of Tierney and Kraus (2013) showed associations between beat-based processing and inhibition. As already mentioned, especially metacognitive knowledge or processes about one’s own cognitive functioning can also serve as a basis from which predictions can be made. It can be assumed that the predictive internal representations of the to be solved rhythm tasks (tapping/ clapping/ drumming along different beats) in the studies of Frischen et al. (2019) and Tierney and Kraus (2013) would stimulate other cognitive processes linked with prediction such as attention and cognitive control (including inhibition). These processes allow participants to constantly compare the actual perceived outcomes with the intended outcomes. Thus, we assume that predictive processes involved in rhythm processing do also stimulate other metacognitive processes which in turn have a facilitating effect on executive functions such as inhibition. This assumption goes well with the finding that children and adults with Attention Deficit Hyperactivity Disorder (ADHD) have problems in synchronizing movements to a beat (Puyjarinet et al., 2017). Since ADHD is associated with poor executive functions, this finding could probably highlight the association between predictive rhythm abilities and executive functions.

Taken together, these studies give a first indication that predictive processes could play a key role in the association between rhythm processing and cognitive-motor abilities. The fact that predictive processes are involved in various abilities across domains points in the direction that this process could serve as a cross-domain mechanism explaining the link between rhythm processing and cognitive-motor abilities. Moreover, the reported studies suggest that predictive processes in general play a key role during early learning and development as it is suggested by several researchers (e.g., Nagai, 2019; Köster et al., 2020). However, we are aware that our assumption is based on only a few studies, and that this idea should be investigated by empirical studies specifically designed for this purpose. Such studies need to assess rhythm processing as such, so that it is controlled to which extent predictive processes are stimulated. This component is missing in many previous studies. Moreover, the study of populations with atypical developmental (or developmental decline in late adulthood) could give more insights in these associations. Specifically, it could be interesting to further investigate the neural processes associated with predictive rhythm processing as demonstrated in children with dyslexia (Colling et al., 2017). Future results from studies with clinical samples showing atypical (or declining) development could provide information on whether the malfunction is only found in predictive rhythm processing or whether prediction is generally affected across domains (as it was addressed by Opitz et al. (2020) in children with DCD). Additionally, further experimental studies could focus on the causal links and investigate whether rhythm training can benefit predictive processes related to rhythm and further predictive processes in other domains. Also, it would be interesting to evaluate whether this also works the other way around (e.g., in how far a training in predictive processes within another domain also improves predictive processes related to musical rhythm).

## Conclusion

With our article we developed and substantiated the hypothesis that predictive processes could be considered as a potential explanation for the link between rhythmic abilities, especially beat-based processing and various cognitive-motor abilities. Because predictive processes are a crucial element of beat-based rhythm processing as well as of cognitive-motor abilities, it is possible that prediction as a cross-domain working principle is a central mechanism to explain the connections between rhythmic abilities and cognitive-motor abilities found in several studies. Our analyses of existing findings on associations between rhythm and language, motor development and executive functions are in line with our assumption. Moreover, results from samples with atypical development indicate that a malfunction in predictive rhythm processing can be associated with significant limitations in cognitive-motor processing. To better investigate these relations, further empirical studies are needed that capture predictive processes while processing rhythms (e.g., through EEG) and cognitive-motor abilities. Randomized controlled studies can give further insights into potential causal relations. Furthermore, future studies could address the question of whether certain rhythms (e.g., highly familiar vs. unfamiliar) or certain meters (e.g., duple meter vs. triple meter) particularly stimulate predictive processes and, for example, examine the extent to which the complexity of a beat-based rhythm matters.

## Data availability statement

The original contributions presented in the study are included in the article/supplementary material, further inquiries can be directed to the corresponding authors.

## Author contributions

UF, FD, and GS contributed to the conception of the manuscript. UF and GS wrote the first draft of the manuscript. All authors contributed to the article and approved the submitted version.

## Conflict of interest

The authors declare that the research was conducted in the absence of any commercial or financial relationships that could be construed as a potential conflict of interest.

## Publisher’s note

All claims expressed in this article are solely those of the authors and do not necessarily represent those of their affiliated organizations, or those of the publisher, the editors and the reviewers. Any product that may be evaluated in this article, or claim that may be made by its manufacturer, is not guaranteed or endorsed by the publisher.

## Glossary

***Beat (Tactus):*** One count of the meter, the most natural rate at which a listener might tap or clap to music.

***Beat-based processing:*** The processing of beat-based stimuli; beat-based stimuli show a regular structure of reoccurring beats.

***Childhood:*** The period from the fifth to the tenth year of life (Schwarzer and Jovanovic, 2015).

***Early childhood:*** The period from the second to the fourth year of life (Schwarzer and Jovanovic, 2015).

***Entrainment:*** A neural process; when neural oscillations couple to an external rhythm.

***Infancy:*** The first year of a child’s life (Schwarzer and Jovanovic, 2015).

***Newborns:*** The first two weeks of life (Schwarzer and Jovanovic, 2015).

***Meter:*** Organizes a series of beats into recurring patterns of stressed and unstressed beats.

***Rhythm:*** Serial pattern of variable tone durations in a melody that unfolds within a timing framework of a recurring pattern of beats.

***Tempo:*** The pace of music, or the rate at which beats unfold over time.
